# Applications of Nanomaterials in Electrogenerated Chemiluminescence Biosensors

**DOI:** 10.3390/s90100674

**Published:** 2009-01-23

**Authors:** Honglan Qi, Yage Peng, Qiang Gao, Chengxiao Zhang

**Affiliations:** Key Laboratory of Analytical Chemistry for Life Science of Shaanxi Province, School of Chemistry and Materials Science, Shaanxi Normal University, Xi'an, 710062, P. R. China

**Keywords:** Electrogenerated Chemiluminescence, Nanomaterials, Biosensor, Amplification, Review

## Abstract

Electrogenerated chemiluminescence (also called electrochemiluminescence and abbreviated ECL) involves the generation of species at electrode surfaces that then undergo electron-transfer reactions to form excited states that emit light. ECL biosensor, combining advantages offered by the selectivity of the biological recognition elements and the sensitivity of ECL technique, is a powerful device for ultrasensitive biomolecule detection and quantification. Nanomaterials are of considerable interest in the biosensor field owing to their unique physical and chemical properties, which have led to novel biosensors that have exhibited high sensitivity and stability. Nanomaterials including nanoparticles and nanotubes, prepared from metals, semiconductor, carbon or polymeric species, have been widely investigated for their ability to enhance the efficiencies of ECL biosensors, such as taking as modification electrode materials, or as carrier of ECL labels and ECL-emitting species. Particularly useful application of nanomaterials in ECL biosensors with emphasis on the years 2004-2008 is reviewed. Remarks on application of nanomaterials in ECL biosensors are also surveyed.

## 1. Introduction

Electrogenerated chemiluminescence (also called electrochemiluminescence and abbreviated ECL) involves the generation of species at electrode surfaces that then undergo electron-transfer reactions to form excited states that emit light [1]. Since the first detailed ECL studies by Kuwana, Hercules and Bard *et al.* in the mid-1960s [2-4], the ECL technique has become a very powerful analytical tool and has been widely used in the areas of, for example, immunoassay, food and water testing, and biowarfare agent detection. ECL detector has also been successfully exploited as a detector in flow injection analysis, high-performance liquid chromatography, capillary electrophoresis, and micro total analysis. Some excellent reviews focused on mechanism, type and its application of ECL were presented from 2004 to 2008 [1, 5-10].

Biosensors are defined as analytical devices incorporating a biological material, a biologically derived material or a biomimic intimately associated with or integrated within a physicochemical transducer or transducing microsystem, which may be optical, electrochemical, thermometric, piezoelectric, magnetic or micromechanical detector [11]. The ECL detection technique has many distinct advantages over other detection techniques [12]. For example, compared with the fluorescence technique, the ECL technique does not involve a light source and, hence, the attendant problems of scattered light and luminescent impurities. Moreover, the specificity of the ECL reaction associated with the ECL label and the coreactant species decreases problems with side reactions and is characterized by good spatial and temporal resolution [1]. Biosensors based on electrogenerated chemiluminescence transducers, combining advantages offered by the selectivity of the biological recognition elements and the sensitivity of ECL technique, are powerful tool for ultrasensitive biomolecule detection and quantification.

Nanomaterials including nanoparticles and nanotubes, prepared from metals, semiconductor, carbon or polymeric species, are of considerable interest in the biosensor field owing to their unique physical and chemical properties, which has led to novel biosensors that have exhibited high sensitivity and stability [13-15]. Particularly, nanomaterials have been investigated for their ability to enhance the efficiencies of ECL biosensors.

The aim of the present review is to give the readers a critical overview of nanomaterials applications in ECL biosensors. For the sake of clarity, this review will specifically focus on the application of nanomaterials in ECL biosensors in view of different functions of nanomaterials on the enhancing ECL signal based on taking as modification electrode materials, carrier of ECL labels and ECL-emitting species. Particular attention will be given to publications that appeared from 2004 to 2008. The remarkable sensitivity of ECL biosensors is achieved by coupling nanomaterial-based amplification units and various amplification processes. The use of nanomaterials carriers for designing multi-target ECL protocols will be documented in detail. Readers are referred to several excellent references [1, 5-10] and relevant websites [16, 17] for further and deeper discussions on certain specific topics.

## 2. Nanomaterials as modification electrode materials

The most important step for building a biosensor is to immobilize the biomolecule on the transducer. A successful platform should have special properties for immobilizing or integrating biomolecule stably at a transducer surface and efficiently maintain the functionality of the biomolecule, while providing accessibility to the target analyte and an intimate contact with the transducer surface. The development of a stable and good biocompatible matrix for immobilization of bimolecules is very crucial to improve the analytical performance of a biosensor. More and more attention has been paid to ECL biosensors functionalized with nanomaterials due to an enormous surface area-to-volume ratio of nanomaterials, and highly susceptibility of ECL to surrounding environments. The diversity in compositions (inorganic or organic, metals or semiconductors), shapes (particles, rods, wires, tubes, etc.), and the readiness for surface functionalization (physical, chemical, or biological) has enabled the fabrication of various functional nanostructures for ECL biosensor. In this section, different nanomaterial-modified electrode interfaces in ECL biosensors, such as gold nanoparticles, carbon nanotubes and other nanoparticle-modified electrodes, are presented.

### 2.1 Metal nanomaterials

Gold nanoparticles (Au NPs), which are stable metal nanomaterials, present fascinating aspects, such as their assembly into multiple types involving materials science, the behavior of the individual particles, size-related electronic, magnetic, and optical properties (quantum size effect), and their applications to catalysis and biology [18]. The unique properties of Au NPs modified electrode interfaces that are different from conventional electrodes lead to novel ECL biosensors with high sensitivity and good stability in immunoassay, DNA assay [19-21]. The enhancement of ECL signals on biosensors with Au NPs modified electrode is mainly attributed to the increase of the surface area.

Dong *et al.* developed an ECL alcohol dehydrogenase (ADH) biosensor, by self-assembling ADH to ruthenium(II) tris(bipyridine) (Ru(bpy)_3_^2+^)-Au NPs aggregates on an indium tin oxide electrode (ITO) surface [19]. Positively charged Ru(bpy)_3_^2+^ was stably immobilized on the electrode surface with negatively charged Au NPs in the form of aggregate via electrostatic interaction. Au NPs are favorable candidates for the immobilization of enzymes because amine groups and cysteine residues in the enzymes are known to bind strongly with Au NPs. Such biosensors combine enzymatic selectivity with the sensitivity of ECL detection for quantification of enzyme substrates with high sensitivity and selectivity.

**Figure 1.**
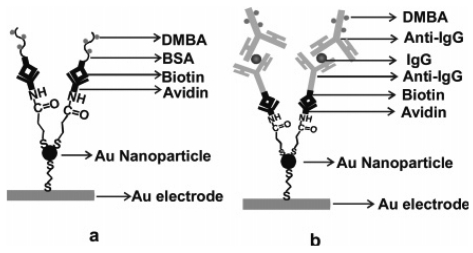
Schematic diagrams of immobilization of BSA (a) and IgG (b) on the gold electrode with gold nanoparticle amplification. Reprinted from Ref [20] with permission from The American Chemical Society.

Besides, Au NPs can act as tiny conduction centers to facilitate the transfer of electrons. Wang and coworkers [20] developed an ECL biosensor for the determination of biological substances including bovine serum albumin and immunoglobulin G (IgG) using 4-(dimethylamino) butyric acid (DMBA) as a label on a gold nanoparticles modified gold electrode. As shown in Figure 1, a gold nanoparticle layer was first combined into the surface of the 2-mm-diameter gold electrode. Avidin was covalently conjugated to a self-assembled monolayer of 3-mercaptopropanoic acid on the gold nanoparticle layer. Biotinylated BSA-DMBA was then immobilized on the gold nanoparticle layer of the gold electrode via the avidin-biotin reaction. IgG was tested via a typical sandwich-type immobilization method. Sensitivity enhancements of 10- and 6-fold were obtained with Au NPs amplification for BAS and IgG over their direct immobilization on an electrode, due to the increase of the electrode area, resulting in the increase of immobilization amount of recognition bimolecular.

The same idea for DNA hybridization detection was reported by Zhang *et al.* [21], in which the surface density of single stranded DNA on the gold nanoparticle modified gold electrode was 12-fold higher than that on the bare gold electrode. Gold nanoparticles were self-assembled on a gold electrode associated with a 1,6-hexanedithiol monolayer. A ruthenium complex served as an ECL tag. Hybridization was induced by exposure of the target ssDNA gold electrode to the solution of ECL probe consisting of complementary ssDNA tagged with ruthenium complex. The detection limit of target ssDNA on a gold nanoparticle modified gold electrode (6.7×10^-12^ M) is much lower than that on a bare gold electrode (1.2×10^-10^ M). Sensitivity enhancements of 18-fold were obtained with Au nanoparticle amplification for DNA over their direct immobilization on an electrode.

Another possible reason for the enhancement of ECL signals on Au NPs modified electrode is the catalytic effect of Au NPs on oxidation/reduction of ECL-emitting species. ECL of luminol [22] and lucigenin [23] on the surface of Au NPs modified gold electrode via a linkage via thiol-gold bonds, ECL of N-(aminobutyl)-N-ethylisoluminol (ABEI) on the surface of Au NPs modified+paraffin-impregnated graphite electrode via physical adsorption [24], and ECL of Ru(bpy)_3_^2+^ [25] on the surface of Au NPs modified ITO via a linkage via thiol-gold bonds, have been reported. At an Au NPs functionalized electrode interface, the sensitivity of ECL signal was much improved compared with conventional electrodes. Up to 5-fold sensitivity enhancements were obtained with Au NPs amplification for ABEI at Au NPs dropped on the surface of a paraffin-impregnated graphite electrode over on a bare electrode [24]. Sensitivity and stability enhancement for Ru(bpy)_3_^2+^ was obtained at ITO electrodes modified with Au NPs by reducing the large overpotential of tripropylamine (TPA) oxidation and reduce a corrosive effect in the ITO surface at high anodic potentials [25]. The size and nature of NPs and the nature of the substrate electrode can also affect the ECL behavior [26]. Besides, other gold nanomaterials, such as gold nanorods, have also been investigated as nanostructures for ECL [27]. The intensities of ECL peaks were enhanced about 2-10-fold on a gold-nanorod-modified gold electrode in neutral solution than at a gold-nanosphere-modified gold electrode.

However, poor stability has been observed at high potential at Au NPs modified electrode, which is due to an oxidation of the thiol layer [21] or the oxidation of gold nanoparticles [20]. Therefore, the first cycle of cyclic voltammetry and the corresponding ECL were recorded [20] or low applied potential was employed [21] in the ECL detection.

Colloidal Ag (Ag NPs) is another important nanomaterial, which possesses tremendous specific surface area, good biocompatibility, and electrical activity [28]. Much stronger ECL emission was found from Ag NPs/Au substrate electrode than that from Au NPs/Au substrate due to its excellent electrical activity [29]. In addition to the most frequently used Au NPs and Ag nanoparticles, other metal nanoparticles, such as platinum nanoparticles, have also been used in the design of nanoparticles functionalized electrode interface for ECL [30].

### 2.2 Carbon nanotubes

Carbon nanotubes (CNT), well-ordered, high aspect ratio allotropes of carbon, whose outstanding properties have sparked an abundance of research since their discovery in 1991 [31], are one of the more popular carbon nanomaterials for ECL biosensors. Single-walled carbon nanotubes (SWNT) are constructed of a single sheet of graphite (diameter 0.4–2 nm), while multi-walled carbon nanotubes (MWNT) consist of multiple concentric graphite cylinders of increasing diameter (2–100 nm). The two main variants, SWNT and MWNT, possess a high tensile strength, are ultra-light weight, and have excellent chemical and thermal stability. In combination with their metallic and semi-conductive electronic properties, this remarkable array of features has seen a plethora of applications proposed [32]. The groups of Wang, Dong, Chen and Lee have done much work on ECL biosensors based on carbon nanotube functionalized electrode interfaces. Recent studies demonstrated that CNT can enhance the electrochemical reactivity [33-35], accumulate important bimolecules [36, 37], and alleviate surface fouling effects [38]. Improvement in sensitivity and stability of ECL biosensors was obtained at the carbon nanotube modified electrode surfaces. Dong reported that the electrochemistry and ECL of Ru(bpy)_3_^2+^ ion-exchanged in CNT/Nafion composite films with TPA as a coreactant at a glassy carbon electrode [35]. It was found that the interfusion of CNT in Nafion resulted in a high peak current of Ru(bpy)_3_^2+^ and high ECL intensity, which indicated that the composite film had more open structures and a larger surface area allowing faster diffusion of Ru(bpy)_3_^2+^ and that the CNT could adsorb Ru(bpy)_3_^2+^ and also acted as conducting pathways to connect Ru(bpy)_3_^2+^ sites to the electrode. Sensitivity enhancement of two and three orders on carbon nanotube modified electrodes was obtained with CNT amplification for TPA over that at a silica/Nafion composite film-modified electrode and that at obtained for pure Nafion films, respectively [35]. To take advantage of these remarkable properties of CNT in ECL biosensor applications, CNT usually need to be properly functionalized and immobilized. Treatment with a nitric acid and sulfuric acid mixture (1:3, v/v) led to multiwall carbon nanotubes terminated with carboxylic acid groups that were usually used in the functionalized of the carbon nanotubes [39].

From the viewpoint of ECL biosensors, effectively immobilizing CNT on the surface of electrode is more significant than that just casting it onto the surface of electrode [39-42], which also can extend their potential applications. The strategy involves the immobilization of CNT on the surface of electrode with Nafion [35, 43-45], Eastman-AQ [34, 46], SiO_2_ [47] and polystyrene (PSP) [48]. An ECL sensor based on PSP with a carbon nanotube composite film was recently developed, in which the PSP was used as an immobilization matrix to entrap the ECL reagent Ru(bpy)_3_^2+^ due to the electrostatic interactions between sulfonic acid groups and Ru(bpy)_3_^2+^ cations. The introduction of CNT into PSP acted not only as a conducting pathway to accelerate the electron transfer but also as a suitable matrix to immobilize Ru(bpy)_3_^2+^ on the electrode by hydrophobic interaction. Furthermore, the results indicated the ECL intensity produced at this composite film was over 3-fold greater compared with that of the pure PSP film due to the electrocatalytic activity of the CNT. Such a sensor was verified by the sensitive determinations of 2-(dibutylamino)ethanol and TPA[48].

Carbon nanotube paste electrodes, like carbon paste electrodes [49], have been applied in ECL sensing for the determination of acephate and dimethoate coupled with capillary electrophoresis (CE) [50]. Ru(bpy)_3_^2+^-immobilized carbon nanotube paste electrode was fabricated by mixing the MWNT powder, Ru(bpy)_3_^2+^ and mineral oil and was electrically heated. This CE–ECL system coupled with heated modified-electrode provides high sensitivity, wide linear range, satisfying linear relationship and excellent reproducibility for the separation and detection of acephate and dimethoate. Recently, room temperature ionic liquids have attracted intensive interest in electrochemistry because of their unique chemical and physical properties such as high chemical and thermal stabilities, a relatively wide potential window and high ionic conductivity [51-53]. Chen [52] developed a composite paste electrode consisting of MWNT and room temperature ionic liquids for fabrication of ECL sensors. These ECL sensors exhibited extraordinary stability during long-term potential cycling and have been developed for determination of methamphetamine hydrochloride.

### 2.3 Other nanomaterials

Other nanomaterials, such as SiO_2_ [54], clay [55], Fe_3_O_4_ [56], and Zeolite Y sieves [57], are also used as electrode modification materials to construct functionalized electrode interfaces for ECL biosensors. These materials have a high chemical inertness and provide a wide range of anode working potentials with low electrical resistivity. For example, an ECL sensor was fabricated by preparing {SiO_2_/ Ru(bpy)_3_^2+^}*n* multilayer films, in which positively charged Ru(bpy)_3_^2+^ and negatively charged SiO_2_ nanoparticles were assembled on ITO electrodes by a layer-by-layer method. Electrochemical and ECL behaviors of the {SiO_2_/Ru(bpy)_3_^2+^}*n* multilayer film-modified electrodes were studied and used for the ECL determination of TPA. The sensitivity was one order of magnitude higher than that observed for previous reported immobilization methods for the determination of TPA [54]. Another ECL sensor obtained by immobilizing Ru(bpy)_3_^2+^ in {clay/Ru(bpy)_3_^2+^}*n* multilayer films by layer-by-layer assembly was investigated by the same group. The stable multilayer films of clay and Ru(bpy)_3_^2+^ were assembled from their aqueous dispersions by alternate adsorption of negatively charged clay platelets and positively charged Ru(bpy)_3_^2+^. The multilayer film modified electrode was used for the ECL detection of TPA and oxalate. The proposed novel immobilized method exhibited good stability, reproducibility and high sensitivity for the determination of TPA and oxalate, which mainly resulted from the contribution of clay nanoparticles with appreciable surface area, special structural features and unusual intercalation properties. The detection limits were 20 nM TPA and 100 nM oxalate, respectively [55]. The ECL sensor was fabricated based on the multilayer films of Nafion-stabilized magnetic nanoparticles (Nafion/Fe_3_O_4_) formed on a platinum electrode surface by means of an external magnet. The ECL sensor based on the Nafion/ Fe_3_O_4_ multilayer films is more sensitive than that based on pure Nafion films, which is maybe due to the fast mass transport in the Nafion/Fe_3_O_4_ multilayer films [56].

## 3. Nanomaterials as carrier of ECL probes

In ECL biosensors, great efforts have been made to improve the sensitivity of ECL biosensor by using multiple ECL labels loaded on metal nanoparticles, carbon nanotubes, microsized polystyrene microspheres and silica NPs externally or internally. That is to say, nanomaterials are used as ECL probe carriers. A large number of ECL labels are encapsulated inside or doped onto single nanoparticles, which produces a strong ECL signal and results in sensitive ECL biosensors.

### 3.1 Metal nanoparticles

**Figure 2.**
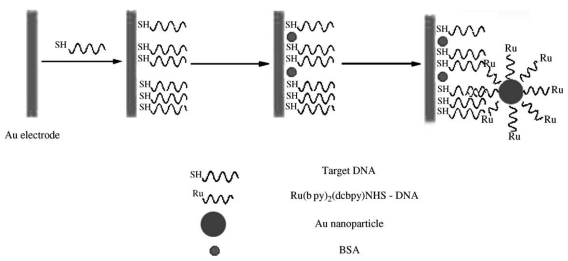
Schematic diagram of the ECL detection for DNA hybridization. Reprinted from Ref [58] with permission from Elsevier.

Metal nanoparticles were also used as ECL carriers in ECL probes for the fabrication of highly sensitive ECL biosensors [58-60]. Zhang, *et. al.* reported an ECL DNA biosensor based on ruthenium bis(2,2′-bipyridine)(2,2′-bipyridine-4,4′-dicarboxylic acid)-*N*-hydroxysuccinimide ester (Ru(bpy)_2_(dcbpy) NHS) used as a ECL label and gold nanoparticles as a carrier [58]. As shown in Figure 2, probe ss-DNA was self-assembled at the 3′-terminal with a thiol group to the surface of gold nanoparticle and covalently labeled at the 5′-terminal of a phosphate group with Ru(bpy)_2_(dcbpy)NHS and the resulting conjugate, (Ru(bpy)_2_(dcbpy)NHS)-ss-DNA-Au, was taken as a ECL probe. When the target analyte ss-DNA was immobilized on a gold electrode by a self-assembled monolayer technique and then hybridized with the ECL probe to form a double-stranded DNA, a strong ECL response was electrochemically generated. The ECL probe of Ru(bpy)_2_(dcbpy)NHS)-ss-DNA-Au employing multiple reporters per hybridization event enhances the ECL detection sensitivity due to its offering a remarkable amplification of hybridization events. A detection limit of 5.0×10^−12^ M for target ss-DNA was achieved with the gold nanoparticle amplification.

Dong *et al.* synthesized Ru(bpy)_3_^2+^-gold nanoparticle aggregates (Ru–AuNPs) via electrostatic interactions by mixing citrate-capped AuNPs and Ru(bpy)_3_Cl_2_ in aqueous medium and following attached the as-formed Ru-AuNPs on a sulfhydryl-derivated ITO electrode surface via Au-S interaction. The Ru–AuNP-modified ITO electrode is quite stable, exhibits excellent ECL behavior, and hence holds great promise for solid-state ECL detection in capillary electrophoresisor a CE microchip [59]. Fang, et.al. fabricated a controllable solid-state Ru(bpy)_3_^2+^-ECL film by immobilization of a Ru(bpy)_3_^2+^-Au nanoparticle composite on a cysteamine - derivatized Au electrode, self-assembled ferrocene-labeled DNA molecular beacon on the resultant electrode via Au-S interaction [60]. Inducement of conformation change of the Fc-MB by hybridization was used as reagentless DNA ECL biosensors to recognize or to detect sequence-specific DNA.

### 3.2 Carbon nanotubes

Carbon nanotubes were also used as ECL carriers in ECL probes for the fabrication of highly sensitive ECL biosensors. SWNT loaded with large of tris(2,2′-bipyridyl) ruthenium derivative tags was synthesized and exhibited excellent ECL signaling ability [61]. Zhang *et al.* developed an ultrasensitive ECL detection method for DNA hybridization based on SWNT carrying a large number of ruthenium complex tags. The probe single ss-DNA and ruthenium complex were loaded at SWNT, which was used as an ECL probe. When the captured ss-DNA with a thiol group was self-assembled onto the surface of gold electrode, and then hybridized with target ss-DNA and further hybridized with the ECL probe to form DNA sandwich conjugate, a strong ECL response was electrochemically generated. SWNT loaded with large of tris(2,2′-bipyridyl) ruthenium derivative tags exhibits excellent ECL signaling ability in the presence of a trace amount of DNA target and the developed ECL method based on the multiple reporters per hybridization event offers a high sensitivity for the detection of DNA hybridization. The ECL intensity was linearly related to the concentration of perfect-matched target ss-DNA in the range from 2.4×10^-14^ to 1.7×10^-12^ M with a detection limit of 9.0×10^-15^ M [61].

### 3.3 Polymeric microbeads

**Figure 3.**
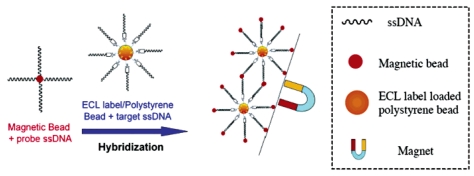
Schematic diagram of DNA hybridization on a polystyrene bead as the ECL label carrier and a magnetic bead for the separation of analyte-contained ECL label/polystyrene beads. Reprinted from Ref [62] with permission from The American Chemical Society.

**Figure 4.**
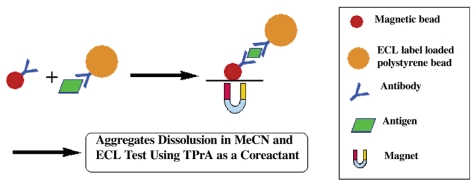
Schematic diagram showing the formation of a sandwich-type aggregate between an antibody-coated MB and an antibody-coated PSB containing entrapped ECL labels in the presence of the antigen species, and the separation of the newly formed aggregate with a magnet as well as the subsequent dissolution and ECL detection in MeCN using TPA as the coreactant. Reprinted from Ref [63] with permission from The American Chemical Society.

Polystyrene microspheres/beads (PSB) have been synthesized as the carrier of a large number of hydrophobic ECL labels [1], namely, tris(2,2′-bipyridyl)ruthenium(II) tetrakis(pentafluorophenyl)borate (Ru(bpy)_3_ [B(C_6_F_5_)_4_]_2_), and demonstrated for an ultrasensitive DNA hybridization detection and CRP immunoassay by Miao [62, 63]. As shown in Figure 3, probe single-stranded DNA (p-ssDNA) was attached to the surface of magnetic beads and hybridized with target-ssDNA (t-ssDNA) with immobilized PSB containing a large number of water insoluble Ru(bpy)_3_[B(C_6_F_5_)_4_]_2_ species (7.5×10^9^ molecules/bead) to form a [(probe ssDNA-MB)/(target ssDNA-PSB)] aggregate. Finally, this aggregate is magnetically separated from the reaction mixture and transferred into a MeCN solution, in which the PSBs dissolve and the ECL label is released. Light emission from the released ECL labels is subsequently measured in MeCN in the presence of TPA at a Pt electrode. A similar approach based on a sandwich type immunoassay can be used for the detection of an antigen (e.g., anti-C-reactive protein, CRP), as displayed in Figure 4. With the above PSB-based “high amplification” technique, a detection limit of 1.0 fM (1.0 × 10^-15^M) for a t-ssDNA was achieved, along with a ∼100-fold improvement in the sensitivity in CRP determination compared to a previously reported surface-immobilized ECL method [12]. The sensitive detection of DNA hybridization was attributed to the attachment of a large number of ECL markers (Ru(bpy)_3_[B(C_6_F_5_)_4_]_2_) per DNA duplex formed. While this technique shows enormous signal amplification compared to the conventional, widely used ECL immunoassay with one label per antibody, it requires the use of an organic solvent, acetonitrile, to release the Ru(bpy)_3_^2+^ and generate ECL. This approach is not generally compatible with current commercial ECL instrumentation. Liposomes (100-nm diameter) containing Ru(bpy)_3_^2+^(bpy)2,2-bipyridine) were prepared as an electrogenerated chemiluminescent tag for a sandwich-type immunoassay of human C-reactive protein, which is based on a similar idea of holding multiple labels in a larger container, allows the assay to be carried out in aqueous solution and has the potential to generate multiple labels after release [64], resulting in a sensitive ECL immunoassay for CRP.

### 3.4 Silica nanoparticles

Recently, silica nanoparticles (silica NPs) have shown their unique properties and been adapted to different applications in the bioanalysis field. Syntheses of dye-doped micrometer-sized silica particles by reverse microemulsion method [65, 66] and the Stober method [67-72] have been reported. Tan's group reported that incorporation of dye molecules inside the silica matrix on the basis of a reverse microemulsion method protects them from the surrounding environment, increases photostability, and provides signal enhancement due to the increase in the number of dye molecules doped per nanoparticle [65, 66]. The Shibata group reported the feasibility of incorporating both hydrophobic and hydrophilic dyes by the Stober method [67]. Different ECL silica NPs carriers for ECL probes with similar composition and functionalization have emerged as a particularly fascinating carrier of ECL probe and attracted widespread interest in biology and medicine. The variety of chemical and physical modifications possible with silica increases its versatility, and its biocompatibility makes it a relatively benign material. Furthermore, the silica NP probe is highly hydrophilic and easy to centrifuge for separation, surface modification, and labeling procedures. The silica NPs exhibit enhanced and controllable mechanical and chemical stability and their porosity can also be easily tailored in terms of pore size and organization. Thus, they are superior to polystyrene latex NP probes, which have significant drawbacks. For instance, the hydrophobic property of polystyrene results in easy agglomeration of NPs in aqueous media, and its density (1.05 g/cm^3^) leads to difficulties in separation from solution after the surface modification and labeling processes [70].

Among various ECL systems, Ru(bpy)_3_^2+^-based ECL biosensors have gained more importance due to its superior properties, including high sensitivity and good stability under moderate conditions in aqueous solution [71]. Recently, various ECL biosensors using Ru(bpy)_3_^2+^doped silica nanoparticles (RuDS nanoparticles) were described for genomic [72], proteomic analysis [73-75], and medical assay [76-78].

**Figure 5.**
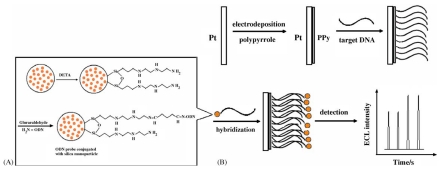
Schematic representation of preparation Ru(bpy)_3_^2+^-doped silica nanoparticles oligonucleotides probes (A) and the electrogenerated chemiluminescence detection of DNA hybridization based on the Ru(bpy)_3_^2+^-doped silica nanoparticles labeled oligonucleotides probes (B). Reprinted from Ref [72] with permission from Elsevier.

Fang *et al.* reported a ECL DNA biosensor with a nanoparticle-amplified response using silica nanoparticles as carriers for Ru(bpy)_3_^2+^ probe [72]. As shown in Figure 5, RuDS nanoparticles were used for DNA labeling with trimethoxysilylpropydiethylenetriamine and glutaraldehyde as linking agents. The RuDS nanoparticle labeled DNA probe was hybridized with target DNA immobilized on the surface of polypyrrole modified Pt electrode. The hybridization events were evaluated by ECL measurements and only the complementary sequence could form a double-stranded DNA with DNA probe and give strong ECL signals. Due to the large number of Ru(bpy)_3_^2+^ molecules inside silica nanoparticle, the assay allows detection at levels as low as 1.0×10^−13^ M of the target DNA.

ECL biosensors with RuDS nanoparticle modified electrode prepared by different methods serving as TPA or polyamine sensors have received much attention [79, 80]. An ECL sensor was developed by coimmobilization of the RuDS nanoparticles and carbon nanotubes on a glassy carbon electrode through hydrophobic interactions [79]. With such a unique immobilization method, a great deal of Ru(bpy)_3_^2+^ was immobilized three-dimensionally on the electrode, which could greatly enhance the ECL response and result in the observed increased sensitivity. On the other hand, CNT played dual roles as matrix to immobilize RuDS nanoparticles and promoter to accelerate the electron transfer between Ru(bpy)_3_^2+^ and the electrode. The as-prepared ECL sensor displayed good sensitivity and stability for the determination of TPA. This sensor shows a detection limit of 2.8 nM for TPA, which is three orders of magnitude lower than that observed at a Nafion-based ECL sensor [82]. However, Ru(bpy)_3_^2+^-doped silica nanoparticles in the above-mentioned films are in random order.

Another strategy for the immobilization of RuDS nanoparticles has been reported by Yang, in which Ru(bpy)_3_^2+^-doped silica nanoparticles were first modified with gold colloids and then immobilized on a 3-mercaptopropyltrimethoxysilane-modified indium tin oxide electrode surface by Au–S interaction [66]. While with such method only one layer of Ru(bpy)_3_^2+^-doped silica nanoparticles could be immobilized, which limits the amount of Ru(bpy)_3_^2+^ effectively involved in ECL reaction., Dong reported an alternative technique for immobilization of RuDS nanoparticles, in which an ECL sensor was fabricated by composing RuDS nanoparticles and Au nanoparticles through layer-by-layer assembly by electrostatic interaction between RuDS nanoparticles and Au nanoparticles [80].

The diameters of most silica coated magnetic nanoparticles and dye-encapsulated silica nanoparticles prepared and studied are larger than 50 nm, and there has been a growing interest in the synthesis of smaller-sized functional magnetic nanoparticles as this would facilitate their applications in biomedical sciences because of their higher surface area and the possibility of larger dye loading. The synthesis and investigation of multifunctional core-shell magnetic silica nanocomposites, with the Fe_3_O_4_ core coated with a silica shell, to which luminescent ruthenium(II) complexes were covalently attached and further encapsulated with an additional layer of silica shell, was reported [82, 83]. These multifunctional nanocomposites were shown to exhibit superparamagnetic behavior, high emission intensity, and electrochemiluminescence. An intense low oxidation potential ECL signal was observed by attachment of these functional nanoparticles onto a fluorosurfactant-modified gold (Aum) electrode *via* application of an external magnetic field [83].

Luminol serving as ECL signal in luminol doped silica nanoparticles was also reported [84-86] for the fabrication of ECL biosensors. However, the application of these ECL biosensors is limited by the consumption of the luminol reagent on the electrode surface.

## 4. Nanomaterials as ECL-emitting species

The sensitivity of ECL biosensor is mainly based on the ECL-emitting species. In the past several years, a number of new ECL-emitting species were synthesized and their ECL properties were investigated. The driving forces behnd these kinds of studies include: (a) finding new luminophores with higher ECL efficiencies and (b) modifying a moiety of the emitter so that it can be used for labeling of bimolecular [1]. Numerous organic and inorganic compounds have been shown to be capable of producing ECL. ECL-emitting species are usually classified into three categories: (a) inorganic systems, which mainly contain organometallic complexes; (b) irganic systems, which cover polycyclic aromatic hydrocarbons and (c), nanomaterial systems. ECL-emitting species discussed in this following section are focused on nanomaterials as ECL-emitting species reported from 2004 to 2008.

### 4.1 Semiconductor nanomaterials

**Figure 6.**
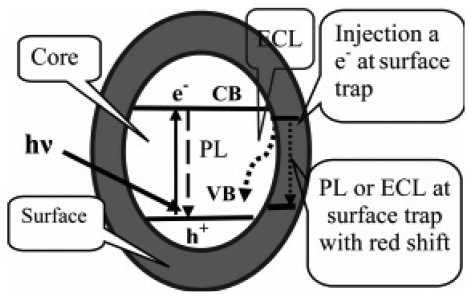
Schematic representations of PL and ECL process of nanoparticle in the core and on the surface. Reprinted from Ref [100] with permission from The American Chemical Society.

In ECL-emitting species, semiconductor nanocrystals (quantum dots) opened a promising field for the development of a new generation of ECL-emitting species. Quantum dots (QDs, also known as nanocrystals, semiconductor nanoparticles or colloidal semiconductor nanocrystals), are roughly spherical and typically have unique optical, electronic and photophysical properties that make them appealing in promising applications in biological labeling, imaging, and detection and as efficient fluorescence resonance energy transfer donors [87]. Since first reported by Bard [88], both elemental semiconductors (e.g., Si and Ge), and many compound semiconductors (e.g., CdS, ZnS, CdSe, and CdTe) were synthesized and found to produce ECL. A number of reviews on the fabrication, properties, and applications of QDs have been reported recently [89-90]. The ECL mechanism of semiconductor NPs follows the general annihilation and coreactant ECL reaction pathways as detailed discussed in Ref [1]. ECL analytical techniques coupled with QDs developed rapidly and were extensively studied in both organic [91-95] and aqueous media [96-103]. Recently, more reports have been focused on ECL of QDs in aqueous solution, which avoids using toxic organic solutions and has potential applications in biological analysis. Zhang *et al.* observed a band gap ECL of ZnS nanoparticles in alkaline aqueous solution at a platinum electrode during the potential applied between -2.0 V (versus Ag/AgCl, saturated KCl) and +0.86 V[100]. It was found that the surface passivation effect and the core/shell structure of ZnS/Zn(OH)_2_ played a significant role in the ECL process and that the similarity of the ECL and PL spectra of semiconductor NPs was dependent on the extent of the surface passivation (schematic representations of the PL and ECL process of nanoparticles in the core and on the surface are shown in Figure 6). The ECL intensity of ZnS NPs in alkaline aqueous solution was greatly enhanced by an addition of K_2_S_2_O_8_ and the ECL intensity linearly increased along with the concentration of ZnS NPs in the range from 1.2×10^-4^ to 1.0×10^-3^ M, which suggests that ZnS NPs in 0.10 M NaOH containing 0.010 M K_2_S_2_O_8_ has potential applications in analytical chemistry. It is noted that the efficiency of the NPs should be improved.

In addition to the solution phase NPs ECL, ECL of NPs films on the surface of electrodes has also received much attention because NPs may find applications in optoelectronic systems or as components in future nanoelectronic devices. Besides, semiconductor NP thin films may also offer better electrochemical and ECL signals, because the solution phase NPs often suffer from low solubility, low concentration, and small diffusion coefficient [1]. Ju *et al.* reported an ECL sensor for the determination of H_2_O_2_ on a CdSe nanocrystal-modified paraffin-impregnated graphite electrode (PIGEs)[97]. The CdSe nanocrystal thin film exhibited two ECL peaks at -1.20 (ECL-1) and -1.50 V (ECL-2) in pH 9.3, 0.1 M PBS during the cyclic sweep between 0 and -1.8 V at 20 mV s-1. ECL-1 showed higher sensitivity to the concentration of oxidant coreactants than ECL-2 and thus was used for ECL detection of the coreactant, H_2_O_2_. A linear response of ECL-1 to H_2_O_2_ was observed in the concentration range of 2.5×10^-7^-6×10^-5^ M with a detection limit of 1.0×10^-7^ M. The fabrication of 10 CdSe nanocrystal thin film modified PIGEs displayed an acceptable reproducibility with a RSD of 1.18% obtained at H_2_O_2_ level of 10 μM.

**Figure 7.**
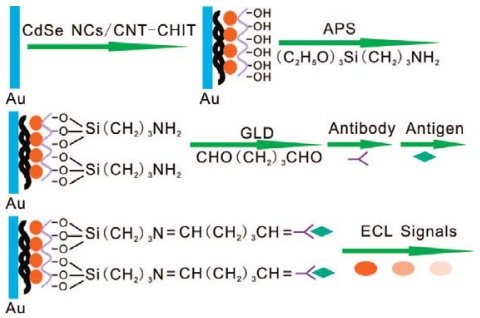
Fabricating Steps of the ECL Immunosensor. Reprinted from Ref [101] with permission from The American Chemical Society.

Zhu *et al.* recently developed a label-free ECL immunosensor for the sensitive detection of human IgG (HIgG) [101]. As shown in Figure 7, an ECL modified electrode was firstly fabricated by covered CdSe nanocrystals, carbon nanotube-chitosan, and 3-aminopropyltriethoxysilane colloidal solution on the Au electrode surface, which showed high ECL intensity and good biocompatibility. The modified electrode was used as an ECL immunosensor for the detection of HIgG after antibody was bound to the functionalized film via glutaric dialdehyde. The specific immunoreaction between HIgG and antibody resulted in the decrease in ECL intensity. The ECL intensity decreased linearly with HIgG concentration in the range of 0.02-200 ng mL^-1^, and the detection limit was 0.001 ng mL^-1^. The immunosensor has the advantages of high sensitivity, speed, specificity, and stability and could become a promising technique for protein detection. It has been suggested that these QDs ECL markers are better labeling agents than commonly used organic dyes.

While ECL research on NPs has focused on the properties of particle ensembles, the exploration of ECL at the single particle level reported allows for the investigation of effects due to particle heterogeneity, which are masked in bulk ECL and EC studies of NPs [104]. For a deep discussion of the electrochemical and ECL behaviors of semiconductor NPs in solutions and in films, readers are referred to very recent comprehensive reviews of this field [1, 105].

### 4.2 Ionic nanomaterials

While most research has focused on inorganic compounds, polymers, or low-molecular weight organic compounds, Recently, a study has been performed on ionic compounds such as [Ru(bpy)_2_(4,4′-(CH_3_(CH_2_)_14_COO)_2_-bpy)](ClO_4_)_2_, the water-insoluble derivative of tris(bipyridine) Ru(II)[106]. The Bard group fabricated nanoscale nanobelts from such an ionic compound by using a simple reprecipitation method. Strong photoluminescence and ECL can be obtained from these in aqueous solution. This is the first report of ECL of a single ionic nanoparticle. These can be useful in the study of the electrochemistry of nanoparticles, as well as the basis of highly sensitive electroanalytical methods, with a small amount of material concentrated on an electrode surface.

## 5. Concluding Remarks

We here have described a variety of ECL biosensors employing nanomaterials as modification electrode materials, carriers of ECL labels and ECL-emitting species. Nanomaterials offer elegant ways for the enhancement of ECL signal and the improvement of sensitivity of ECL biosensors. Such coupling of high sensitivity and stability capabilities permits ECL biosensors to rival the most advanced electrochemical and optical protocols in bioassays. Although completely nanomaterial-based ECL biosensors showed improved ECL characteristics, new materials and immobilization methods are still needed in order to improve the sensitivity and the long-term stability of ECL biosensors.

The combination of the ECL technique with other techniques could lead to the development of new instruments and provide valuable insights into molecular structures and intracellular components of biorelated species [1]. In particular, the integration of nanotechnology, microfabrication techniques and miniaturized devices with novel biochemical detection methodologies should lead to very sensitive and fast assays. With further understanding of ECL mechanisms, new highly efficient and tunable ECL systems, both emitters and coreactants, will be developed for further improving the analytical performance of ECL biosensors.
